# Age-Stratified Analysis of Blood–Brain Barrier Proteins Identifies Lower Occludin Abundance in the 3xTg-AD Model

**DOI:** 10.3390/ijms27198705

**Published:** 2026-09-29

**Authors:** Karen Michelle Delgado-Minjares, Catalina Flores-Maldonado, Jessica Paulina Campos-Blázquez, Sofía Y. Díaz-Miranda, Jesús Andrade-Guerrero, Wendolin Colín-García, Karen León-Arcia, Daniel Martinez-Fong, Rubén G. Contreras, Luis O. Soto-Rojas

**Affiliations:** 1 Departamento de Fisiología, Biofísica y Neurociencias, Centro de Investigación y de Estudios Avanzados del Instituto Politécnico Nacional, Ciudad de México C.P. 07360, Mexico; km95.delgado@gmail.com (K.M.D.-M.); catalina.flores@cinvestav.mx (C.F.-M.); jessica.campos@cinvestav.mx (J.P.C.-B.); daniel.martinezfong@cinvestav.mx (D.M.-F.); 2 Laboratorio de Investigación en Neurociencias, Envejecimiento y Neurodegeneración (LINEN), Carrera de Médico Cirujano, Facultad de Estudios Superiores Iztacala, Universidad Nacional Autónoma de México, Ciudad de México C.P. 54090, Mexico; jesusandrade1007@gmail.com (J.A.-G.); wcg0871@gmail.com (W.C.-G.); klarci@comunidad.unam.mx (K.L.-A.); 3 Departamento de Neurobiología del Desarrollo y Neurofisiología, Instituto de Neurobiología, Universidad Nacional Autónoma de México, Querétaro C.P. 76230, Mexico; yoldi@unam.mx; 4 Programa de Posgrado en Ciencias Biológicas, Unidad de Posgrado, Edificio A, Primer Piso, Circuito de Posgrado, Ciudad Universitaria, Coyoacán, Ciudad de México C.P. 04510, Mexico

**Keywords:** Alzheimer’s disease, mouse model, capillary brain proteins, endothelial cells, tight junctions

## Abstract

Alzheimer’s disease (AD) is a progressive neurodegenerative disorder in which blood–brain barrier (BBB) dysfunction may arise early. We performed an age-stratified, cross-sectional analysis of pericyte- and endothelial-associated markers and junctional proteins (claudin-1, -3, -5, occludin, and VE-cadherin) in cerebral capillary-enriched fractions from male Non-Tg and 3xTg-AD mice at asymptomatic, early, and advanced stages (3, 6, 12, and 16 months of age). Protein abundance was assessed by Western blot, and BBB permeability to 3–5 kDa FITC-dextran was evaluated at 3 months in an independent cohort. At 3 months, the pSRC/SRC ratio and an MMP-9-immunoreactive band were also evaluated in a separate cohort as candidate signaling markers related to occludin dysregulation. We detected no significant age or genotype effects for the pericyte- and endothelial-associated markers or for most junctional protein outcomes. Occludin was the only protein outcome with a significant overall genotype effect, with the largest mean reductions observed at 3 (~80%) and 6 months (~68%). A small exploratory cohort showed a higher hemisphere-derived permeability index in 3xTg-AD mice at 3 months. Neither pSRC/SRC nor the abundance of the MMP-9-immunoreactive band differed significantly between genotypes. These findings identify lower occludin abundance as the principal BBB-associated protein alteration in the 3xTg-AD model and provide exploratory evidence of increased BBB permeability at 3 months. These independent observations warrant longitudinal and translational evaluation to determine whether occludin abundance and BBB permeability could serve as indicators of early neurovascular dysfunction in AD.

## 1. Introduction

Alzheimer’s disease (AD) is the most common type of dementia, characterized by progressive neuronal loss in the hippocampus and other specific brain regions, leading to cognitive and functional decline [1]. Despite more than a century since its first description, the precise mechanisms underlying AD onset and progression remain incompletely understood [2,3]. Several hypotheses have been proposed to explain disease development; however, in recent years, attention has increasingly focused on the vascular contribution to AD pathogenesis. In this context, the two-hit vascular hypothesis has gained particular relevance. This hypothesis proposes that vascular risk factors induce early BBB disruption (hit 1), impairing Aβ clearance and facilitating Aβ accumulation (hit 2), thereby promoting neurodegeneration and AD progression. This hypothesis positions BBB dysfunction as an early event that may contribute to disease initiation rather than solely representing a consequence of advanced neurodegeneration [4].

The BBB is a highly selective neurovascular interface located primarily at brain capillaries and regulates substance exchange between the blood and the brain parenchyma. Specialized endothelial cells form it, supported by pericytes, the basement membrane, astrocyte endfeet, and other cellular components, each of which performs an essential function in maintaining brain homeostasis [5]. Multiple lines of evidence indicate that the BBB and its cellular and molecular components are compromised in AD. At the cellular level, pericytes play a critical role in maintaining BBB integrity, regulating endothelial cell stability, cerebral blood flow, and Aβ clearance through phagocytic and transport mechanisms [6]. Post-mortem studies in AD patients have reported pericyte loss, which correlates with BBB breakdown and cognitive decline [7,8,9]. In parallel, reduced immunoreactivity for endothelial markers, including platelet endothelial cell adhesion molecule-1 (PECAM-1) and cluster of differentiation 34 (CD34), has been reported in AD brains [10,11]. However, reduced marker expression should not necessarily indicate endothelial cell loss, as it may also reflect phenotypic or functional alterations.

A key functional property of brain endothelial cells is their ability to restrict paracellular transport through tight junctions (TJ). The main TJ protein families expressed in the BBB include claudins, occludin, tricellulin, junctional adhesion molecules (JAMs), and zonula occludens (ZO) [12,13,14]. Claudins differ in their permeability properties and tissue distribution; therefore, these proteins can be classified as barrier- (claudin-1, -3, -5) or pore-forming (claudin-12) [15]. Claudin-5 is highly expressed in brain endothelial cells and constitutes a major barrier-forming component that restricts the passage of small molecules across the BBB [13,14]. Claudin-1 has been reported under specific physiological and pathological conditions, including injury and aging, and has been associated with changes in BBB permeability [16,17,18]. Claudin-3 has been considered a barrier-associated claudin; however, evidence from knockout models, transcriptomic studies, and tissue-localization analyses indicates that its contribution to the BBB remains controversial and that claudin-3 is more consistently expressed in choroid plexus epithelial cells at the blood–cerebrospinal fluid barrier (BCSFB) than in parenchymal brain endothelial cells [19,20].

Occludin was the first TJ-associated transmembrane protein discovered and is an integral component of endothelial junctional complexes, although it is not indispensable for TJ strand formation [21,22,23]. Occludin regulates TJ function, and its role is proposed to be tissue-specific [24]. Rather than acting solely as a structural seal, occludin participates in intracellular signaling, protein trafficking, and the dynamic regulation of paracellular permeability [23]. In the brain, occludin downregulation is associated with increased endothelial permeability, edema, inflammation, and neuronal damage [23].

In addition to TJ, endothelial cells also express adherens junction (AJ) proteins, which provide mechanical stability to the endothelium and are essential for proper TJ assembly and maintenance [25]. Vascular endothelial-cadherin (VE-cadherin) is a crucial AJ transmembrane protein predominantly expressed in endothelial cells and plays a key role in maintaining cell–cell adhesion and regulating vascular barrier integrity [26,27]. Under inflammatory conditions, a disintegrin and metalloproteinase 10 (ADAM10) can cleave VE-cadherin, generating an ~90 kDa fragment associated with endothelial junction remodeling and barrier dysfunction [28].

Matrix metalloproteinase-9 (MMP-9) is implicated in BBB disruption. Experimental studies outside AD have associated MMP-9 activation with tight-junction disorganization and occludin loss or degradation [29,30]. SRC proto-oncogene, a non-receptor tyrosine kinase (SRC), can also regulate BBB permeability by phosphorylating and redistributing junctional proteins, including occludin and VE-cadherin, with secondary effects on claudin-5 [26,31,32,33]. Despite their importance, only a limited number of TJ and AJ proteins have been evaluated across multiple ages in AD models [34,35].

Human studies investigating BBB alterations in AD are limited by their predominant focus on late disease stages and by the lack of comprehensive analyses of temporal changes across different BBB components. Notably, only one study has evaluated the progressive reduction in claudin-5 and occludin throughout AD progression [35]. Understanding temporal changes in BBB components is essential for identifying early disease biomarkers and for developing targeted interventions to maintain or restore BBB function. Consequently, experimental models that recapitulate the temporal progression of AD are required to address these gaps.

The triple transgenic AD murine model (3xTg-AD) progressively develops Aβ and tau pathology [36]. At 3 months of age, these mice are considered to be in an asymptomatic-like or preclinical stage and exhibit no evident cognitive deficits [37]. By 6 months of age, corresponding to an early disease stage, they develop Aβ plaques, tau hyperphosphorylation, neuroinflammation, and mild-to-moderate deficits in spatial and working memory [37,38,39]. Advanced stages occur at 12 and 16 months of age, when cortical amyloid deposition is prominent, tau pathology spreads, and severe behavioral deficits, including motor dysfunction and cognitive impairment, are observed [37,38,39].

Previous studies in the 3xTg-AD model have evaluated selected BBB-associated alterations, including changes in the cortical basement membrane, astrocyte endfeet, and collagen IV in the hippocampal fissure, as well as increased gadolinium-based permeability in the *Cornu Ammonis* regions and dentate gyrus [39,40,41]. Nevertheless, these investigations have generally focused on individual BBB domains rather than providing an integrated temporal assessment of cellular markers and endothelial junctional proteins.

To our knowledge, no previous study has compared pericyte- and endothelial-associated markers alongside multiple TJ and AJ proteins in cerebral capillary-enriched fractions across several ages of the 3xTg-AD model. This age-stratified approach can identify protein-specific patterns of vulnerability or preservation, but the cross-sectional design cannot demonstrate within-animal temporal change. The asymptomatic stage of AD is a crucial opportunity for intervention, as neuropathological alterations begin years before cognitive symptoms appear [42]. Identifying early BBB-associated molecular differences may help prioritize mechanisms for longitudinal and functional validation.

Therefore, this study characterized age-stratified changes in key cellular and junctional BBB proteins in the 3xTg-AD model across 3, 6, 12, and 16 months. At 3 months, when we observed the largest descriptive reduction in occludin abundance within the significant overall genotype effect, we further examined BBB permeability and candidate signaling alterations involving pSRC/SRC and MMP-9. This integrated approach was designed to determine whether BBB disruption in 3xTg-AD mice is associated with selective molecular alterations and whether these changes are accompanied by early functional evidence of neurovascular impairment.

## 2. Results

### 2.1. Verification of Cerebral Capillary Enrichment

To evaluate alterations in BBB-related protein abundance in the 3xTg-AD model, we first verified successful enrichment of cerebral capillaries obtained from cortical and subcortical brain regions, using a previously described isolation protocol [43].

**Figure 1:**
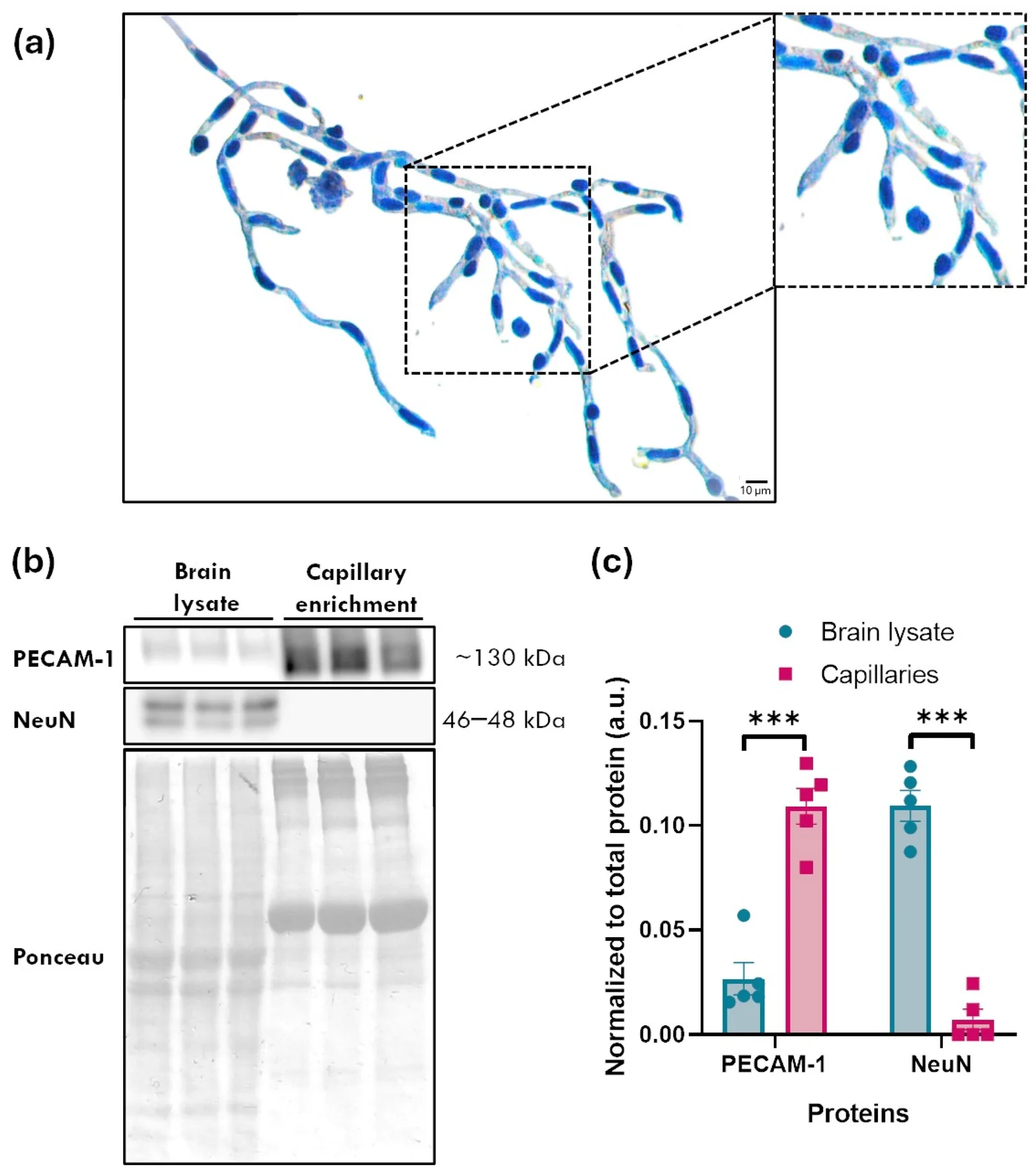
Verification of cerebral capillary enrichment. (**a**) Representative bright-field micrograph of a cerebral capillary-enriched fraction stained with trypan blue, showing tubular microvascular structures with diameters of less than 10 μm. Scale bar: 10 µm. (**b**) Representative Western blot and (**c**) densitometric analysis of PECAM-1 and NeuN in total brain lysates and cerebral capillary-enriched fractions obtained from two independent groups of 3-month-old Non-Tg mice, with Ponceau staining shown as the total-protein loading control. Individual animals are shown together with the mean ± SEM (*n* = 5 animals per group; *** *p* < 0.0001; two-tailed Welch’s *t*-test). Brightness and contrast adjustments were applied uniformly across the representative images and did not alter the underlying data. Abbreviations: a.u., arbitrary units; NeuN, neuronal nuclear antigen; PECAM-1, platelet endothelial cell adhesion molecule-1; SEM, standard error of the mean.

We initially assessed microvascular structures by bright-field microscopy after trypan blue staining. Tubular structures with diameters < 10 μm were observed, consistent with the characteristic morphology of cerebral capillaries (Figure 1a).

After morphological verification, the endothelial marker PECAM-1 and neuronal marker neuronal nuclear antigen (NeuN) were analyzed by Western blot in total brain lysates and cerebral capillary-enriched fractions to assess endothelial enrichment and neuronal contamination (Figure 1b,c).

PECAM-1 was detected in both the brain lysate and the capillary-enriched fraction. The capillary-enriched fraction showed a markedly stronger immunoreactive band at the expected molecular weight of ~130 kDa. Consistent with this observation, normalized PECAM-1 levels were significantly higher in the capillary-enriched fraction than in the total brain lysate (*p* < 0.0001; Figure 1b,c).

Furthermore, NeuN was readily detected in the total brain lysate but was nearly undetectable in the capillary-enriched fraction, resulting in a significant reduction relative to the brain lysate (*p* < 0.0001; Figure 1c). The characteristic microvascular morphology, PECAM-1 enrichment, and marked NeuN depletion support successful, reproducible recovery of a cerebral capillary-enriched fraction with markedly reduced neuronal signal.

### 2.2. No Significant Genotype or Age Effects on Pericyte- and Endothelial-Associated Markers

**Figure 2:**
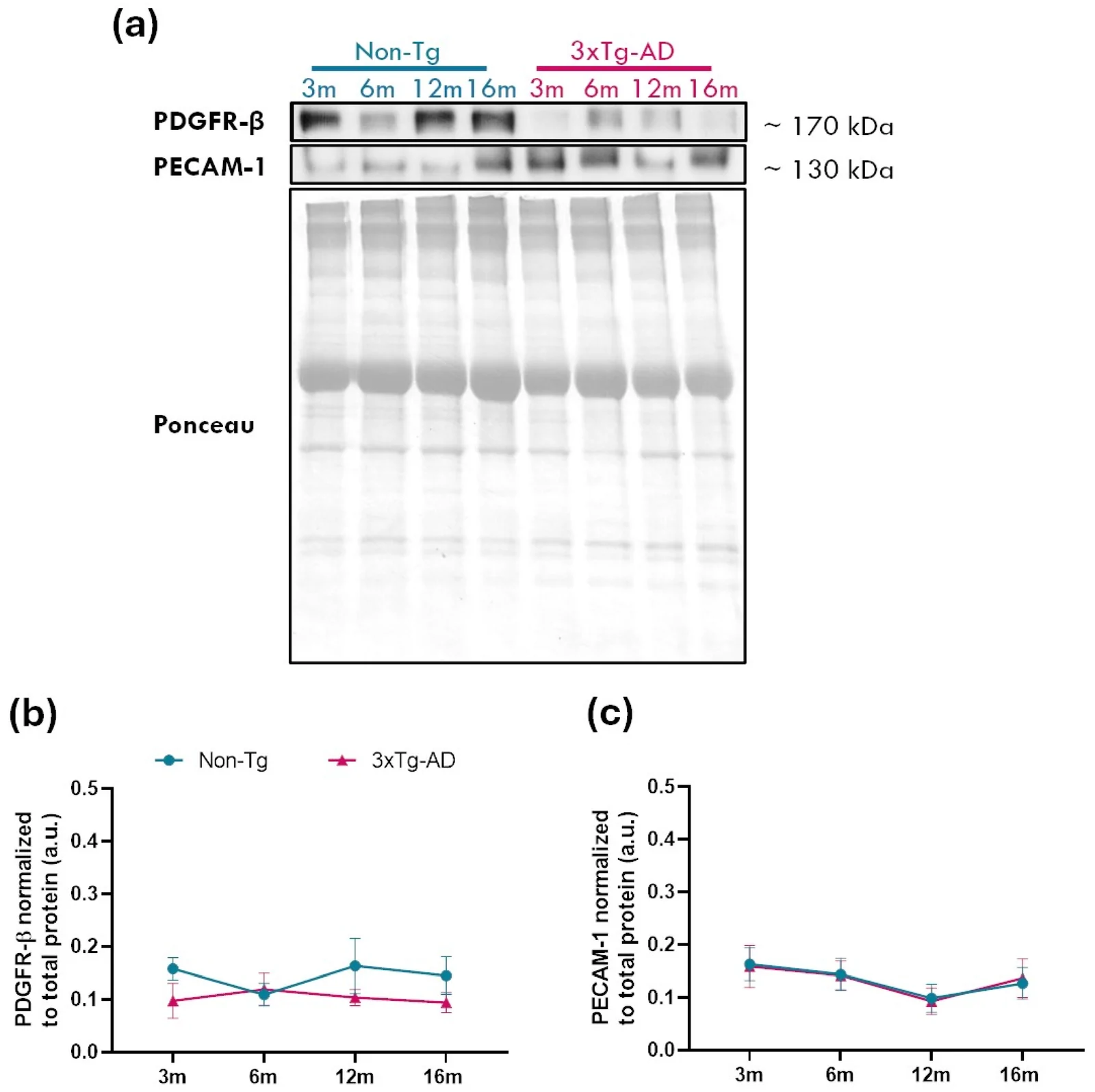
No significant genotype, age, or genotype × age effects on the pericyte-associated marker PDGFR-β or the endothelial marker PECAM-1 in the 3xTg-AD model. (**a**) Representative Western blot images with Ponceau staining as the total-protein loading control; (**b**) PDGFR-β and (**c**) PECAM-1 densitometry in Non-Tg and 3xTg-AD mice at 3, 6, 12, and 16 months. Data are presented as mean ± SEM (*n* = 5 per group; complete dataset). Supplementary Tables S12–S14 report the complete two-way ANOVA, panel-level FDR, and age-specific effect estimates. Abbreviations: 3xTg-AD, triple transgenic Alzheimer’s disease; a.u., arbitrary units; ANOVA, analysis of variance; FDR, false discovery rate; Non-Tg, non-transgenic; PDGFR-β, platelet-derived growth factor receptor beta; PECAM-1, platelet endothelial cell adhesion molecule; SEM, standard error of the mean.

To determine whether the abundance of proteins associated with pericytes and endothelial cells differed across the examined ages of the 3xTg-AD model, the pericyte marker platelet-derived growth factor receptor beta (PDGFR-β) and the endothelial cell marker PECAM-1 were evaluated in the cerebral capillary-enriched fraction (Figure 2).

In the complete dataset, PDGFR-β abundance showed no significant genotype effect after panel-level false discovery rate (FDR) correction (*q* = 0.1807) and no significant age or genotype × age interaction effects (Figure 2a,b; Supplementary Tables S12–S14).

PECAM-1 abundance likewise showed no significant genotype effect after panel-level FDR correction (*q* = 0.9813) and no significant age or genotype × age interaction effects (Figure 2a,c; Supplementary Tables S12–S14).

### 2.3. Selective Reduction in Occludin Among the Evaluated Tight-Junction Proteins in 3xTg-AD Mice

**Figure 3:**
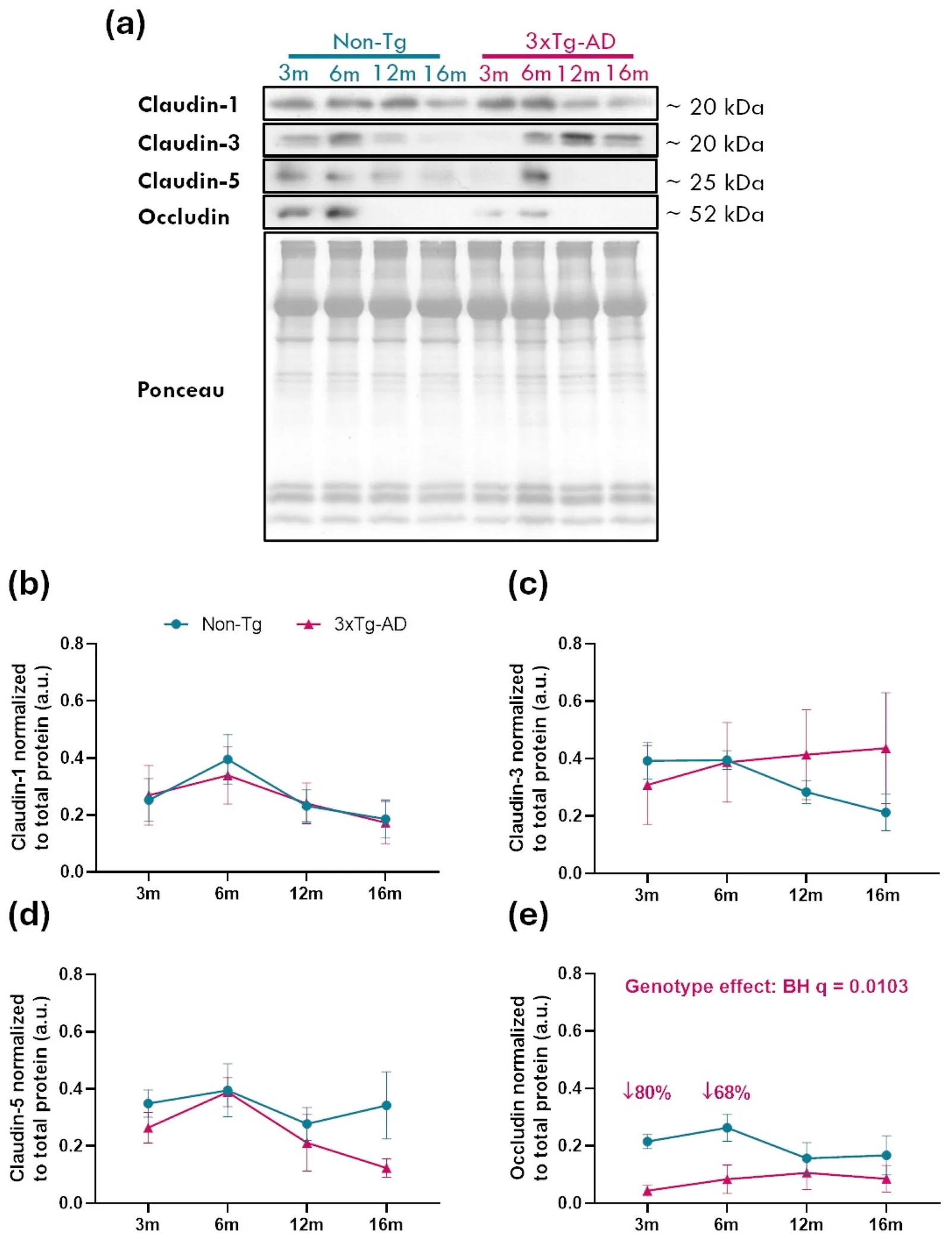
Occludin is selectively reduced in 3xTg-AD mice among the evaluated tight-junction proteins. (**a**) Representative Western blot images of claudin-1, claudin-3, claudin-5, and occludin with Ponceau staining as the total-protein loading control; corresponding densitometry of (**b**) claudin-1, (**c**) claudin-3, (**d**) claudin-5, and (**e**) occludin in Non-Tg and 3xTg-AD mice at 3, 6, 12, and 16 months. Data are presented as mean ± SEM (*n* = 5 per group; complete dataset, including Dixon-flagged observations). Complete-data two-way ANOVA followed by Benjamini–Hochberg correction across the eight overall genotype effects identified occludin as the only tight-junction protein with a significant overall reduction in 3xTg-AD mice (*q* = 0.0103). Complete analyses and Dixon-excluded sensitivity analyses are reported in Supplementary Tables S12–S16. Abbreviations: 3xTg-AD, triple transgenic Alzheimer’s disease; a.u., arbitrary units; ANOVA, analysis of variance; Non-Tg, non-transgenic; SEM, standard error of the mean.

Claudin-1 and claudin-3 showed no significant genotype effects after panel-level FDR correction and no significant age or genotype × age interaction effects in the complete dataset (Figure 3a–c; Supplementary Tables S12–S14). Claudin-5 likewise showed no significant panel-adjusted genotype effect (*F*(1,32) = 3.187, *p* = 0.083706, *q* = 0.1807), and neither age nor the genotype × age interaction was significant. At 16 months, the claudin-5 comparison was nominally significant before multiplicity correction (unadjusted *p* = 0.0454) but not after Šídák adjustment (adjusted *p* = 0.1697; Figure 3a,d; Supplementary Table S14).

Occludin showed a significant overall genotype effect in the complete dataset, with lower abundance in 3xTg-AD mice than in Non-Tg controls (*F*(1,32) = 12.446, *p* = 0.001291, *q* = 0.0103, partial η^2^ = 0.280). Neither age (*F*(3,32) = 0.423, *p* = 0.737625) nor the genotype × age interaction (*F*(3,32) = 0.882, *p* = 0.460732) was significant. The largest between-genotype differences were observed at 3 and 6 months, with mean occludin levels ~80% and 68% lower, respectively, in 3xTg-AD mice than in age-matched Non-Tg controls. Although the corresponding unadjusted *p*-values were 0.0178 (3 months) and 0.0133 (6 months), neither age-specific contrast remained significant after Šídák adjustment (adjusted *p* = 0.0692 and 0.0523, respectively; Figure 3a,e; Supplementary Tables S12–S14). In sensitivity analyses excluding Dixon-flagged observations, both 3 and 6 months reached significance after Šídák adjustment (adjusted *p* = 0.0434 and 0.0110, respectively; Supplementary Table S16).

### 2.4. No Significant Genotype or Age Effects on the Evaluated VE-Cadherin Immunoreactive Forms

**Figure 4:**
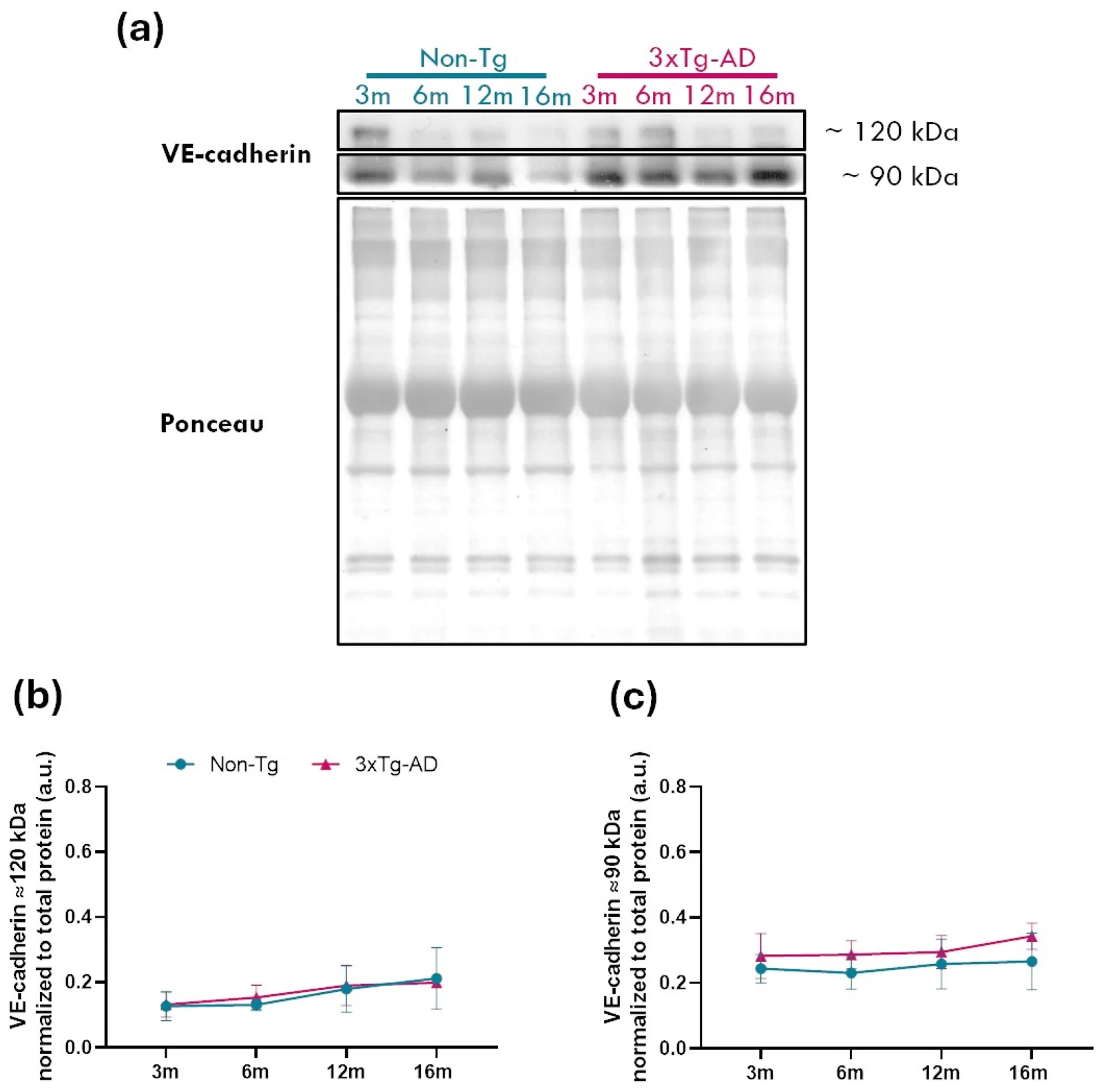
No significant genotype, age, or genotype × age effects on the evaluated VE-cadherin-immunoreactive bands in the 3xTg-AD model. (**a**) Representative Western blot images showing VE-cadherin-immunoreactive bands at ~120 and 90 kDa, with Ponceau staining as the total-protein loading control; corresponding densitometry of (**b**) the ~120 kDa band consistent with full-length VE-cadherin and (**c**) the ~90 kDa lower-molecular-weight band in Non-Tg and 3xTg-AD mice at 3, 6, 12, and 16 months. Data are presented as mean ± SEM (*n* = 5 per group; complete dataset, including Dixon-flagged observations). The ~90 kDa outcome was analyzed on the log10 scale. Complete analyses are reported in Supplementary Tables S12–S14. Abbreviations: 3xTg-AD, triple transgenic Alzheimer’s disease; a.u., arbitrary units; ANOVA, analysis of variance; Non-Tg, non-transgenic; SEM, standard error of the mean.

To assess AJ protein abundance across the examined ages of the 3xTg-AD model, VE-cadherin was quantified in the cerebral capillary-enriched fraction. Two immunoreactive bands were detected at ~120 and 90 kDa and quantified separately (Figure 4a).

The ~120 kDa band, consistent with full-length VE-cadherin, showed no significant genotype effect after panel-level FDR correction (*q* = 0.9813) and no significant age or genotype × age interaction effects in the complete dataset (Figure 4a,b; Supplementary Tables S12–S14).

The ~90 kDa lower-molecular-weight VE-cadherin-immunoreactive band likewise showed no significant genotype effect after panel-level FDR correction (*q* = 0.1807) and no significant age or genotype × age interaction effects (Figure 4a,c; Supplementary Tables S12–S14).

### 2.5. Exploratory Evidence of Increased BBB Permeability at 3 Months in the 3xTg-AD Model

BBB permeability was assessed at 3 months using intraperitoneal 3–5 kDa FITC-dextran in an independent cohort. This age is commonly used to approximate an asymptomatic-like stage in this model and was when the largest descriptive between-genotype difference in occludin abundance was observed.

**Figure 5:**
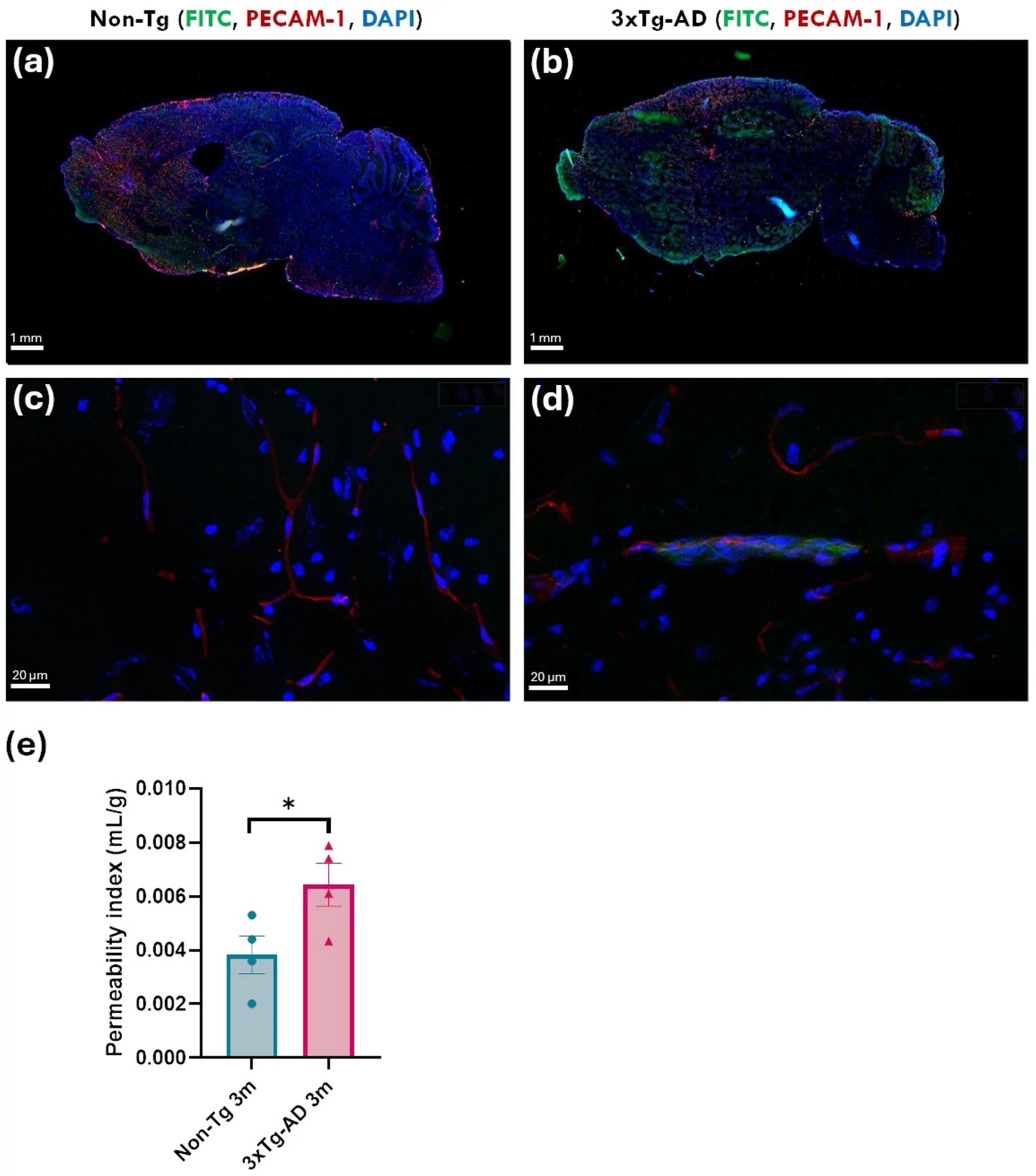
Exploratory evidence of a higher BBB permeability index at 3 months. Representative epifluorescence images show FITC-dextran signal (green), PECAM-1-labeled vessels (red), and nuclei (blue): (**a**) panoramic Non-Tg section, (**b**) panoramic 3xTg-AD section, (**c**) higher-magnification hippocampal region in Non-Tg, and (**d**) the corresponding hippocampal region in 3xTg-AD. The images were used only for qualitative anatomical visualization. Scale bars: 1 mm for panoramic images and 20 µm for higher-magnification images. (**e**) Hemisphere-derived brain permeability index. Individual animals are shown as blue dots (Non-Tg) and red triangles (3xTg-AD), with the mean ± SEM (*n* = 4 per genotype). The unpaired two-tailed Student’s *t*-test yielded a nominal, * *p* = 0.0494, statistically significant; mean difference (3xTg-AD minus Non-Tg), 0.00261 mL/g (95% CI, 0.000009 to 0.005201); Cohen’s d = 1.74. Abbreviations: 3xTg-AD, triple transgenic Alzheimer’s disease; a.u., arbitrary units; DAPI, 4′,6-diamidino-2-phenylindole; FITC, fluorescein isothiocyanate; Non-Tg, non-transgenic; PECAM-1, platelet endothelial cell adhesion molecule; SEM, standard error of the mean.

Qualitative examination of sagittal sections illustrated the anatomical distribution of FITC-dextran signal in the olfactory bulb, prefrontal cortex, and hippocampus (Figure 5a–d).

The hemisphere-derived permeability index was higher in 3xTg-AD mice than in age-matched Non-Tg controls (nominal *p* = 0.0494). The mean difference (3xTg-AD minus Non-Tg) was 0.00261 mL/g (95% CI, 0.000009 to 0.005201), with Cohen’s d = 1.74 (Figure 5e). Given the small cohort, this finding is considered exploratory.

### 2.6. pSRC/SRC Ratio and MMP-9-Immunoreactive Band Abundance Do Not Differ Between Genotypes at 3 Months

**Figure 6:**
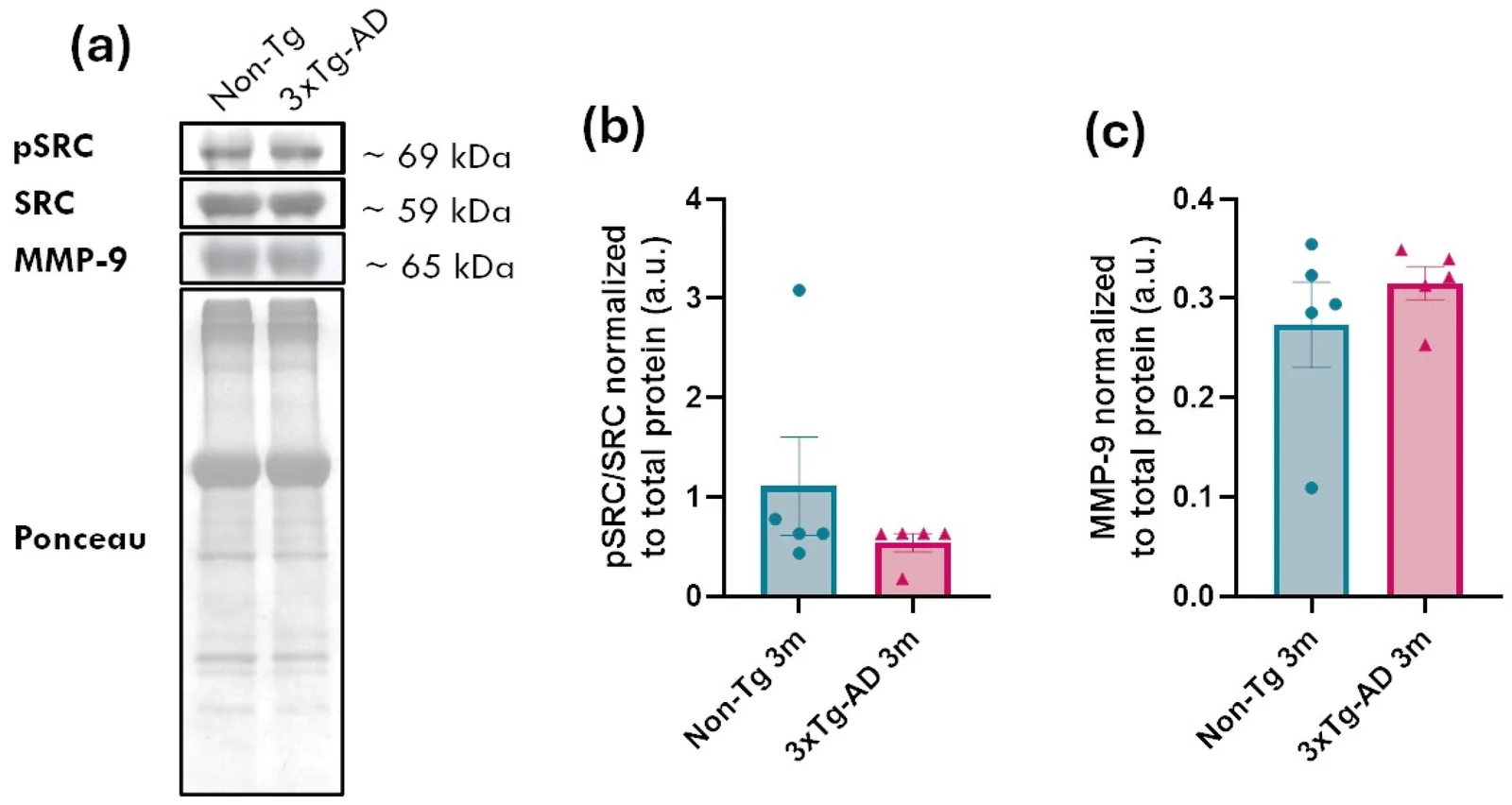
No significant genotype differences in the pSRC/SRC ratio or the MMP-9-immunoreactive band at 3 months in the 3xTg-AD model. (**a**) Representative Western blot images with Ponceau staining as the total-protein loading control; complete-data quantification, including Dixon-flagged observations, of (**b**) pSRC/SRC and (**c**) the ~65 kDa MMP-9-immunoreactive band in Non-Tg and 3xTg-AD mice. Individual animals are shown as blue dots (Non-Tg) and red triangles (3xTg-AD), with the mean ± SEM (*n* = 5 per genotype). Comparisons used unpaired two-tailed Welch *t*-tests. pSRC/SRC: *p* = 0.316; MMP-9-immunoreactive band: *p* = 0.404. Complete-data and Dixon-excluded sensitivity analyses are reported in Supplementary Table S17. Abbreviations: 3xTg-AD, triple transgenic Alzheimer’s disease; a.u., arbitrary units; Non-Tg, non-transgenic; MMP-9, Matrix metalloproteinase-9; pSRC, phosphorylated SRC; SRC, SRC proto-oncogene, non-receptor tyrosine kinase; SEM, standard error of the mean.

Because the largest descriptive between-genotype occludin difference and the exploratory permeability finding occurred at 3 months, pSRC/SRC and the ~65 kDa MMP-9-immunoreactive band were evaluated as candidate signaling markers at this age in an independent cohort (Figure 6a).

In complete-data Welch tests, the pSRC/SRC ratio did not differ significantly between genotypes (*t*(4.266) = 1.134, *p* = 0.316; mean difference, Non-Tg minus 3xTg-AD = 0.571; 95% CI, −0.793 to 1.935; Hedges’ g = 0.65; Figure 6b). The ~65 kDa MMP-9-immunoreactive band also did not differ significantly (*t*(5.195) = −0.909, *p* = 0.404; mean difference, Non-Tg minus 3xTg-AD = −0.0417; 95% CI, −0.158 to 0.075; Hedges’ g = −0.52; Figure 6c). The Dixon-excluded sensitivity analyses yielded the same overall interpretation (Supplementary Table S17).

## 3. Discussion

This is the first age-stratified, targeted study comparing pericyte-associated, endothelial, tight-junction, and adherens-junction proteins in cerebral capillary-enriched fractions from asymptomatic-like, early, and advanced ages in the 3xTg-AD model.

After panel-level FDR control, the principal protein finding was a selective overall reduction in occludin abundance in 3xTg-AD mice, whereas claudin-5 and the other evaluated cellular and junctional outcomes showed no significant FDR-adjusted genotype effects. The largest between-genotype decrease in occludin was observed at 3 (~80%) and 6 (~68%) months, although neither age-specific contrast remained significant after Šídák adjustment in the primary complete-data analysis. In the Dixon-excluded sensitivity analysis, the 3- and 6-month contrasts reached significance after Šídák adjustment, indicating that age-specific inference is sensitive to individual observations. The 3-month permeability analysis, performed in a separate small cohort, provided exploratory evidence of BBB dysfunction, whereas neither the MMP-9-immunoreactive band nor the pSRC/SRC ratio differed between genotypes.

PDGFR-β and PECAM-1 did not show significant genotype, age, or genotype × age effects. These null abundance results should not be interpreted as evidence that pericyte number, pericyte coverage, endothelial cell density, or vascular function are preserved. We did not assess regional and zonation-related heterogeneity of pericytes and endothelial cells [44,45], and pericyte impairment and reduced PDGF-BB/PDGFR-β signaling have been identified in AD [46,47,48].

At the TJ level, claudin-1 abundance showed no significant genotype- or age-dependent differences. Claudin-1, although poorly studied in AD, increases in response to injury and aging and has been associated with BBB permeability [16,17,18]. Across ages, claudin-1 abundance showed modest variability without a consistent genotype-related pattern. These findings do not support a measurable change in total claudin-1 abundance in the 3xTg-AD model, although localization or function were not assessed.

Claudin-3 also showed no significant genotype, age, or genotype × age effects. However, this result requires particular caution. Current evidence indicates that claudin-3 is robustly expressed in choroid plexus epithelial cells at the blood–CSF barrier but is absent or minimally expressed in parenchymal brain endothelial cells; its endothelial expression remains controversial [19]. Because the choroid plexus was not removed before preparation of the capillary-enriched fraction, the detected signal cannot be attributed exclusively to cerebral endothelial cells or interpreted as evidence of BBB endothelial claudin-3 expression. Thus, these data are not BBB-specific and may include blood–CSF-barrier components carried through the enrichment procedure [19,49].

Claudin-5, the predominant barrier-forming claudin in cerebral endothelial cells [14], did not show a significant effect of age or genotype × age interaction, and its overall genotype effect did not remain significant after panel-level FDR correction. At 16 months, mean claudin-5 abundance was lower in 3xTg-AD mice than in age-matched Non-Tg controls; however, this age-specific difference did not remain significant after Šídák adjustment. Thus, the present data do not provide statistical evidence for a late-stage reduction in total claudin-5 abundance. Nevertheless, regionally restricted alterations cannot be excluded, as such changes may be diluted in whole-brain analyses or remain below the detection sensitivity of this approach [50].

Among the evaluated tight-junction proteins, occludin therefore emerged as the most selectively altered component in the 3xTg-AD model. The more pronounced differences observed at earlier ages warrant particular interest, although the present cross-sectional design does not establish an age-specific or progressive trajectory. Occludin is dynamically regulated through phosphorylation, ubiquitination, internalization, recycling, and degradation, processes that influence its junctional localization and paracellular barrier function [23,35,51,52,53,54]. Aβ exposure has been shown to reduce occludin expression and increase permeability in human brain endothelial cells [51], providing a plausible mechanistic framework for further investigation. However, because occludin abundance and permeability were assessed in independent cohorts, the present study does not establish an individual-level association or causal relationship between these findings.

We evaluated MMP-9 and SRC as candidate pathways associated with occludin dysregulation. MMP-9 activity has been implicated in tight-junction disorganization and occludin loss or degradation [29,30,55,56], whereas SRC activation can promote occludin phosphorylation and junctional destabilization [31,32]. However, neither the ~65 kDa MMP-9-immunoreactive band nor the pSRC/SRC ratio differed significantly between genotypes. Although the ~65 kDa band represents an MMP-9-immunoreactive species, its molecular weight and immunoreactivity do not establish its molecular identity, processing state, or catalytic activity [57,58]. Thus, the null band-abundance result does not exclude changes in MMP-9 activity, secretion, localization, processing, or regulation by endogenous inhibitors. Likewise, an unchanged pSRC/SRC ratio cannot exclude transient or spatially restricted SRC activation. These pathways remain untested mechanistic possibilities rather than explanations demonstrated by the present data. Other mechanisms, including oxidative stress, MAPK signaling, post-translational modifications, and altered occludin trafficking, also warrant direct investigation [51,52,53,54,59,60].

Neither the ~120 kDa full-length VE-cadherin form nor the ~90 kDa immunoreactive band differed significantly between genotypes or across ages. A soluble VE-cadherin fragment near this molecular-weight range has been described under inflammatory conditions [28], but molecular weight alone does not establish in vivo cleavage or fragment identity in the present samples. We therefore interpret the ~90 kDa result only as a lower-molecular-weight VE-cadherin-immunoreactive band.

The exploratory permeability difference at 3 months is consistent with evidence that BBB dysfunction can occur early in AD progression [4,61], but it does not establish causality or an individual-level relationship with occludin. Because permeability and occludin were measured in separate cohorts, their co-occurrence at the same nominal age reflects only stage-level concordance. A prospectively powered replication, paired assessment of permeability and junctional proteins in the same animals, and direct manipulation or localization of occludin are needed to determine whether the two observations are reproducibly or mechanistically related.

Several limitations should be considered. The study included only male mice; it was not powered to evaluate sex interactions, and the findings cannot be generalized to females. The age-stratified design was cross-sectional. Mixed cortical and subcortical capillary-enriched fractions may obscure regional effects, and the preparation was not a purified endothelial fraction. Because the choroid plexus was not removed and its carryover was not quantified, claudin-3 cannot be assigned specifically to BBB endothelium. Western blotting measured total abundance but not cellular localization, junctional organization, vascular coverage, or ultrastructure. The permeability experiment was limited by its small sample size and the absence of prospectively standardized regional imaging. The modest sample size and the lack of significant age-specific occludin contrasts after primary-analysis multiplicity adjustment limit temporal localization of the overall genotype effect, despite significance at 3 and 6 months in the Dixon-excluded sensitivity analyses. Immunoblotting alone cannot establish the molecular identity, processing state, or catalytic activity of the ~65 kDa MMP-9-immunoreactive band; an orthogonal identification method and gelatin zymography or another activity-based assay would be required. Future studies should include both sexes, region-resolved localization and ultrastructure, prospectively powered paired permeability protein measurements, quantitative imaging, and direct mechanistic assays of occludin regulation.

## 4. Materials and Methods

### 4.1. Mouse Model and Experimental Design

The experimental model used was the 3xTg-AD, which carries the human *APPSwe* and *MAPTP301L* transgenes on a homozygous *PSEN1M146V* knock-in background. These genetic alterations are associated with progressive Aβ and tau pathology [36]. Animals were housed under standard conditions with 12 h light–dark cycles, a temperature of 22 ± 2 °C, and a relative humidity of 60 ± 5%, with food and water available ad libitum.

This study used male 3xTg-AD mice and age-matched Non-Tg controls (129C57/BL6 background). Dr. Sofía Yolanda Díaz Miranda of the Institute of Neurobiology (INB)-UNAM provided the animals. All experimental procedures were approved by the Institutional Committee for the Care and Use of Laboratory Animals of the INB (project number 117.A, date of approval: 24 July 2023) and were conducted in accordance with the Mexican Official Standard NOM-062-ZOO-1999. The study is reported in accordance with the ARRIVE 2.0 guidelines. The study was designed a priori as an initial age-stratified characterization in one sex and was not powered to test sex × genotype × age interactions; consequently, inference is limited to males.

We distributed 68 male mice across four independent experimental sets. First, we verified the cerebral capillary-enrichment procedure using two independent groups of 3-month-old Non-Tg mice (*n* = 5 per group). One group provided total brain lysates, whereas the second group provided cerebral capillary-enriched fractions. We also used the latter group for bright-field morphological verification of the enriched fraction. We assessed endothelial enrichment and neuronal contamination by Western blot analysis of PECAM-1 and NeuN, respectively, by comparing total brain lysates with cerebral capillary-enriched fractions from these independent groups.

Second, we evaluated the age-stratified abundance of BBB-associated proteins in Non-Tg and 3xTg-AD mice at 3, 6, 12, and 16 months of age. These ages are commonly used to approximate asymptomatic-like, early, and advanced phases of the model, but the present cross-sectional design did not independently establish disease stage in each animal. We included five animals in each genotype and age group, resulting in eight experimental groups and a total of 40 mice. These animals were used to evaluate PDGFR-β, PECAM-1, claudin-1, claudin-3, claudin-5, occludin, and VE-cadherin abundance in cerebral capillary-enriched fractions.

Third and fourth, based on the age-stratified analysis, subsequent experiments focused on 3 months, when the largest complete-data mean difference in occludin was observed. BBB permeability was evaluated in one independent cohort (*n* = 4 per genotype), whereas another independent cohort (*n* = 5 per genotype) examined pSRC/SRC and the ~65 kDa MMP-9-immunoreactive band. Both cohorts were independent of each other and of the 40 animals used for protein-abundance analysis. The individual animal was the experimental unit.

This stage-focused exploratory design examined functional permeability and candidate pathways at the age of the largest observed complete-data occludin mean difference. BBB permeability was evaluated in a small exploratory cohort; this experiment was not prospectively powered for confirmatory inference.

### 4.2. Isolation and Verification of the Cerebral Capillary-Enriched Fraction

We isolated cerebral capillaries using previously published protocols, with minor modifications [43,62]. We verified the reproducible enrichment of cerebral microvessels before the downstream protein analyses. Accordingly, the resulting preparation is referred to throughout the manuscript as a cerebral capillary-enriched fraction rather than as purified cerebral capillaries.

Euthanasia was performed by decapitation, and brains were rapidly removed, snap-frozen on dry ice, and stored at −80 °C until further processing for capillary isolation.

To reduce capillary binding, all tubes used during the isolation were incubated overnight at 4 °C with 1% bovine serum albumin (BSA). Cortical and subcortical regions were homogenized in Dulbecco’s Modified Eagle Medium (DMEM) using a glass tissue homogenizer with 10 gentle strokes without rotating the pestle. The choroid plexus was not systematically removed before homogenization. Therefore, residual choroid-plexus material was not quantified and cannot be excluded from the resulting fraction. The homogenate was centrifuged at 2050× *g* for 5 min at 4 °C, and the supernatant was carefully removed by vacuum aspiration. The pellets were resuspended in 25% dextran (D8821-50G, Sigma-Aldrich, Saint Louis, MO, USA) prepared in Dulbecco’s phosphate-buffered saline (DPBS) and centrifuged at 10,000× *g* for 15 min at 4 °C. The supernatant was carefully aspirated without disturbing the pellet or introducing myelin and cellular debris.

The resulting pellet was resuspended in DPBS, transferred to a 40 μm cell strainer, and washed thoroughly with DPBS. The strainer was then inverted and rinsed with DMEM supplemented with 0.5% BSA to obtain a fraction enriched in cerebral capillaries.

For morphological verification, 10 μL of the capillary-enriched fraction was mixed with 10 μL of trypan blue, and the samples were examined under the inverted bright-field microscope to confirm the presence and characteristic tubular morphology consistent with cerebral capillaries. The representative microphotograph presented in this manuscript was adjusted for brightness and contrast.

Because the previously published protocols had already characterized pericyte- and astrocyte-associated components within the resulting fraction [43,62], molecular verification in the present study focused on confirming endothelial enrichment and excluding neuronal contamination. PECAM-1 was evaluated as an endothelial marker, whereas NeuN was used as a neuronal marker, as described in Section 4.4.

### 4.3. Protein Extraction and Quantification from Cerebral Capillary-Enriched Fractions

The capillary-enriched fraction was centrifuged at 5000× *g* for 10 min at 4 °C, and the supernatant was carefully discarded. The pellet was resuspended in ice-cold lysis buffer containing 50 mM HEPES, 1% Triton X-100, 0.5% sodium deoxycholate, 0.1% sodium dodecyl sulfate, 500 mM NaCl, 10 mM magnesium chloride, 1 mM sodium orthovanadate, and 1 mM sodium fluoride, supplemented with cOmplete^TM^ protease inhibitor cocktail (Roche, cat. no. 04693116001, Mannheim, BW, Germany).

The samples were sonicated on ice using an ultrasonic homogenizer (Vibra-Cell, cat. no. 432-0132, Newtown, CT, USA) with three 5 s pulses at 50% amplitude to disrupt cells and solubilize proteins. Lysates were then centrifuged at 10,000× *g* for 10 min at 4 °C to remove insoluble material, and the resulting supernatants were collected for downstream analysis. Protein concentration was determined using the Pierce™ BCA Protein Assay Kit (Thermo Fisher Scientific, cat. no. 23225, Rockford, IL, USA), according to the manufacturer’s instructions. Protein extracts from each animal were processed and analyzed independently, without pooling, and each animal represented one biological replicate.

### 4.4. Western Blot Analysis

Protein extracts from cerebral capillary-enriched fractions (20 µg per sample) were separated by molecular weight using sodium dodecyl sulfate–polyacrylamide gel electrophoresis (SDS-PAGE) on 10% or 15% polyacrylamide gels, depending on the target protein’s molecular weight, at 110 V for 2 h. Proteins were then transferred onto nitrocellulose membranes (Bio-Rad, cat. no. 1620112, Hércules, CA, USA) using a wet transfer system (Bio-Rad, cat. no. 1703930) with ice-cold transfer buffer (25 mM Tris-base, 192 mM glycine, and 20% methanol) at 100 V for 2 h.

After transfer, the membranes were briefly stained with Ponceau to verify protein transfer efficiency and equal loading, then blocked with 1% BSA in Tris-buffered saline containing 0.1% Tween-20 (T-TBS) for 1 h at room temperature. Membranes were incubated overnight at 4 °C with primary antibodies against PDGFR-β (rabbit; Abcam; cat. no. ab32570, Cambridge, UK; 1:500), PECAM-1 (goat; R&D Systems; cat. no. AF3628, Minneapolis, MN, USA; 1:2000), NeuN (mouse; Sigma-Aldrich; cat. no. MAB377; 1:6000), claudin-1 (mouse; Thermo Fisher Scientific; cat. no. 37-4900, Rockford, IL, USA; 1:500), claudin-3 (rabbit; Thermo Fisher Scientific; cat. no. 34-1700; 1:500), claudin-5 (rabbit; Abcam; cat. no. ab15106; 1:2000), occludin (mouse; Thermo Fisher Scientific; cat. no. 33-1500; 1:500), VE-cadherin (rabbit; Thermo Fisher Scientific; cat. no. 36-1900; 1:500), MMP-9 (goat; R&D Systems; cat. no. AF909; 1:500), total SRC (mouse; Thermo Fisher Scientific; cat. no. AHO1152; 1:500) and phosphorylated (pSRC; rabbit; Thermo Fisher Scientific; cat. no. 44-660G; 1:500).

After primary antibody incubation, membranes were washed with T-TBS and incubated with species-appropriate horseradish peroxidase (HRP)-conjugated secondary antibodies (1:10,000) for 2 h at room temperature. Immunoreactive bands were visualized, and exposure conditions were selected to avoid signal saturation.

Densitometric analysis was performed on raw, non-saturated images using ImageJ software (version 1.54; https://imagej.net/ij/; [accessed on 28 September 2026]). For each membrane, we measured the integrated density of the target immunoreactive band after background subtraction and normalized it to the Ponceau-stained membrane, which represents the total protein content in the corresponding lane. We applied the same region-of-interest criteria to all samples analyzed on the same membrane. We normalized the pSRC and total SRC signals to the corresponding total Ponceau S lane signal before calculating the pSRC/SRC ratio. The ~65 kDa MMP-9-immunoreactive band was quantified as an antibody-reactive species; molecular weight and immunoreactivity alone were not used to assign catalytic activity or definitive proteolytic state.

Representative Western blot images were selected from samples with normalized values closest to the median of their respective groups. Any brightness or contrast adjustments were performed only after densitometric analysis, were applied uniformly to the entire image, and did not alter the relative intensity of individual bands.

### 4.5. Dextran-Based Blood–Brain Barrier Permeability Assay

BBB permeability was assessed using low-molecular-weight FITC-dextran (Sigma-Aldrich, cat. no. FD4) with a nominal molecular weight of 3–5 kDa, following a previously published protocol with minor modifications [63]. This tracer was selected to detect subtle increases in BBB permeability to low-molecular-weight solutes. Each mouse received 100 µL of a 2 mM FITC-dextran solution, corresponding to 0.2 µmol and approximately 0.6–1.0 mg per animal based on the nominal 3–5 kDa molecular-weight range.

Five minutes after administration, animals were anesthetized i.p. with Ketamine (100 mg/kg) and Xylazine (5 mg/kg). At 20 min after FITC–dextran administration, blood was collected, and the animals were transcardially perfused with DPBS to remove circulating intravascular tracer before brain collection.

Blood samples were collected before transcardial perfusion, and serum was isolated by centrifugation at 10,000× *g* for 10 min at 4 °C for FITC-dextran fluorescence measurement. The brains were then removed and divided into two hemispheres. One hemisphere was homogenized in DPBS and centrifuged at 15,000× *g* for 20 min at 4 °C. The resulting supernatant was used for quantitative analysis of FITC-dextran fluorescence in brain tissue.

Measurements were performed using 20 μL of serum or brain homogenate diluted in 30 μL of DPBS. DPBS alone was used as a fluorescence blank. Fluorescence intensity was measured using a Tecan Infinite 200 microplate reader (Tecan Life Science, Männedorf, ZH, Switzerland) at 490 nm excitation and 520 nm emission, and was expressed as relative fluorescence units (*RFUs*).

The permeability index (*PI*) was calculated after subtraction of DPBS background fluorescence using the following formula:

$$\mathrm{PI}\;(\mathrm{mL}/g)=\frac{\left(\frac{\mathrm{Tissue}\;\mathrm{RFUs}}{g\;\mathrm{tissue}\;\mathrm{weight}}\right)}{\left(\frac{\mathrm{Serum}\;\mathrm{RFUs}}{\mathrm{mL}\;\mathrm{serum}}\right)}$$

The contralateral hemisphere was processed for immunofluorescence to visualize the anatomical distribution of FITC-dextran signal. Tissues were embedded in Tissue-Tek O.C.T. compound (Sakura, cat. no. 4583, Torrance, CA, USA) and sectioned at a thickness of 20 µm. Sections were fixed in methanol for 15 min at room temperature, then washed with DPBS containing 0.05% Triton X-100. Non-specific binding was blocked with 0.5% BSA for 1 h at room temperature in a humidified chamber. Sections were then incubated overnight at 4 °C in a humidified chamber with a primary antibody against PECAM-1 (goat; R&D Systems; cat. no. AF3628; 1:200).

After washing, sections were incubated with a tetramethylrhodamine isothiocyanate (TRITC)-conjugated anti-goat secondary antibody (Thermo Fisher Scientific, cat. no. A16101; 1:100) for 1 h at room temperature. Autofluorescence was reduced with a saturated solution of Sudan Black for 10 min, followed by washing with DPBS. Nuclei were counterstained with 4′,6-diamidino-2-phenylindole (DAPI, Sigma-Aldrich, cat. num. D9542), and slides were mounted using VECTASHIELD (Vector Laboratories, cat. num. H-100, Newark, CA, USA). Sagittal brain sections were digitized at 40× magnification using a KF-PRO-400 slide scanner (Konfoong Bioinformation Tech Co., Ltd., Yuyao City, ZJ, China), and PECAM-1 immunoreactivity was visualized using QuPath software (v0.6.0). The representative panoramic brain microphotographs presented in this manuscript were adjusted for brightness and contrast. Microscopy was used exclusively for qualitative anatomical visualization.

### 4.6. Statistical Analysis

Data are presented as mean ± SEM, with each animal treated as an independent biological replicate. Complete datasets were used for all primary analyses. We used the Shapiro–Wilk test to assess normality of model residuals. Potential outliers were assessed using the two-sided Dixon r10 Q test. For groups with *n* = 5, a significance level of α = 0.05 was used, corresponding to a critical value of Qcrit = 0.710 according to standard Dixon Q-test tables [64]. Dixon-flagged observations were retained in the primary analyses and excluded only in the corresponding sensitivity analyses. Given the small sample size, we evaluated model assumptions using residual and graphical diagnostics rather than formal within-cell normality tests. Claudin-3 and the ~90 kDa VE-cadherin outcome were log10-transformed before ANOVA to improve model assumptions.

Cerebral capillary-enrichment measurements from independent brain-lysate and capillary-enriched groups were compared using unpaired two-tailed Welch *t*-tests.

Age-stratified protein-abundance data were analyzed using ordinary two-way ANOVA, with genotype and age as between-subject factors and the genotype × age interaction included in the model. No matching or repeated-measures structure was specified because different animals were evaluated at each age. To control multiplicity across the panel, Benjamini–Hochberg FDR correction (*q* < 0.05) was applied to the eight overall genotype effects: PDGFR-β, PECAM-1, claudin-1, claudin-3, claudin-5, occludin, and the ~120 and 90 kDa VE-cadherin outcomes. Age-specific genotype contrasts were treated as follow-up tests only for outcomes passing the FDR screen and were Šídák-adjusted across four ages. All age-specific contrasts, mean differences with 95% confidence intervals, and Hedges’ g values are reported for transparency in the Supplementary Materials. Sensitivity analyses that became unbalanced after exclusion of Dixon-flagged observations were analyzed using ordinary two-way ANOVA with Type III sums of squares; the same panel-level FDR and Šídák follow-up framework was applied, and these analyses were interpreted exclusively as sensitivity analyses.

BBB permeability at 3 months was compared using an unpaired two-tailed Student’s *t*-test and was considered exploratory because of the small sample size (*n* = 4 per genotype) and absence of an independent replication. The between-genotype mean difference with 95% confidence interval and Cohen’s d were also calculated; the *p*-value is reported as nominal. Complete-data pSRC/SRC and ~65 kDa MMP-9-immunoreactive-band comparisons used unpaired two-tailed Welch *t*-tests; Dixon-excluded analyses were reported only as sensitivity analyses, with mean differences, 95% confidence intervals, and Hedges’ g provided in the Supplementary Materials.

Data are presented as mean ± SEM. Figure 2, Figure 3 and Figure 4 show group summary data, whereas Figure 1, Figure 5 and Figure 6 display individual observations where applicable. Statistical analyses and visualization were performed in GraphPad Prism version 8.0 and independently reproduced from the individual-level values in Python 3.12 with SciPy 1.17.0. All tests were two-sided. Statistical significance was set at *p* < 0.05 for single comparisons, *q* < 0.05 for the panel-level FDR screen, and adjusted *p* < 0.05 for follow-up contrasts.

## 5. Conclusions

This study identifies occludin as a priority molecular candidate for understanding alterations in BBB integrity in the 3xTg-AD model. Its selective reduction, together with preliminary evidence of increased BBB permeability at an early disease stage, supports the translational evaluation of occludin-centered signatures in longitudinal human studies, including biofluids and vascular-derived biomarkers. Establishing whether these alterations can detect early BBB involvement before advanced neurodegeneration could open new opportunities for neurovascular biomarkers and BBB-targeted intervention strategies in Alzheimer’s disease.

## Data Availability

The original contributions presented in this study are included in the article/Supplementary Materials. Further inquiries can be directed to the corresponding authors.

## Supplementary Materials

The following supporting information can be downloaded at: https://www.mdpi.com/article/10.3390/ijms27198705/s1.

## Abbreviations

The following abbreviations are used in this manuscript:

3xTg-AD	Triple-transgenic Alzheimer’s disease
Aβ	Amyloid-beta
AD	Alzheimer’s disease
ADAM10	A disintegrin and metalloproteinase 10
AJ	Adherens junction
ANOVA	Analysis of variance
a.u.	Arbitrary units
BBB	Blood–brain barrier
BCSFB	Blood–cerebrospinal fluid barrier
BSA	Bovine serum albumin
CD34	Cluster of differentiation 34
CI	Confidence interval
DAPI	4′,6-Diamidino-2-phenylindole
DMEM	Dulbecco’s Modified Eagle Medium
DPBS	Dulbecco’s phosphate-buffered saline
FDR	False discovery rate
FITC	Fluorescein isothiocyanate
HRP	Horseradish peroxidase
INB	Institute of Neurobiology
i.p.	Intraperitoneally
JAMs	Junctional adhesion molecules
MAPK	Mitogen-Activated Protein Kinase
MMP-9	Matrix metalloproteinase-9
NeuN	Neuronal nuclear antigen
Non-Tg	Non-transgenic
PECAM-1	Platelet endothelial cell adhesion molecule-1
PDGFR-β	Platelet-derived growth factor receptor beta
*PI*	Permeability index
pSRC	Phosphorylated SRC
*RFUs*	Relative fluorescence units
SEM	Standard error of the mean
SDS-PAGE	Sodium dodecyl sulfate–polyacrylamide gel electrophoresis
SRC	SRC proto-oncogene, non-receptor tyrosine kinase
T-TBS	Tris-buffered saline containing 0.1% Tween-20
TJ	Tight junction
TRITC	Tetramethylrhodamine isothiocyanate
VE-cadherin	Vascular endothelial cadherin
ZO	Zonula occludens
