# Supplementary material for: Age-Stratified Analysis of Blood–Brain Barrier Proteins Identifies Lower Occludin Abundance in the 3xTg-AD Model
Source: Int J Mol Sci. 2026 Sep 29;27(19):8705. doi: 10.3390/ijms27198705 (PMC13665031; doi:10.3390/ijms27198705)
Supplement: Supplementary file 1 [file ijms-27-08705-s001.zip › KMDM_LOSR_FINAL_Supplementary.pdf]

Supplementary Data

Age-Stratified Analysis of Blood–Brain Barrier Proteins Identifies Lower Occludin Abundance in the 3xTg-AD Model

Statistical framework. Complete datasets are prioritized. Potential outliers were identified using Dixon's two-sided r10 Q test (n = 5, α = 0.05, Qcrit = 0.710) and retained in all primary analyses; Dixon-flagged observations were excluded only in sensitivity analyses. We analyzed age-stratified protein outcomes using ordinary two-way ANOVA, with genotype and age as between-subject factors and the genotype × age interaction included in the model; we did not specify matching or a repeated-measures structure because different animals were evaluated at each age. Panel-level multiplicity was controlled using the Benjamini-Hochberg false discovery rate (FDR) across eight overall genotype effects. Age-specific genotype contrasts were treated as follow-up comparisons for outcomes passing the panel-level FDR screen and were adjusted across four ages using Šídák's procedure; all age-specific contrasts are reported for transparency. Claudin-3 and the approximately 90 kDa VE-cadherin outcome were log10-transformed before ANOVA to improve model assumptions. Sensitivity analyses that became unbalanced after exclusion of Dixon-flagged observations were analyzed using ordinary two-way ANOVA with Type III sums of squares.

Table S1. PDGFR-beta individual values and Dixon Q audit

Group	Sorted individual values	Minimum	Maximum	Qmin	Qmax	Qcrit alpha 0.05	Flagged for sensitivity
**Non-Tg 3m**	0.099069, 0.131488, 0.160724, 0.172091, 0.226785	0.099069	0.226785	0.2538	0.4282	0.7100	No
**Non-Tg 6m**	0.059859, 0.078343, 0.088214, 0.154572, 0.165744	0.059859	0.165744	0.1746	0.1055	0.7100	No
**Non-Tg 12m**	0.011874, 0.084173, 0.175551, 0.250627, 0.295142	0.011874	0.295142	0.2552	0.1571	0.7100	No
**Non-Tg 16m**	0.041706, 0.076360, 0.196164, 0.203363, 0.209002	0.041706	0.209002	0.2071	0.0337	0.7100	No
**3xTg-AD 3m**	0.025441, 0.031291, 0.089349, 0.147826, 0.194860	0.025441	0.194860	0.0345	0.2776	0.7100	No
**3xTg-AD 6m**	0.039409, 0.085617, 0.105009, 0.150618, 0.216721	0.039409	0.216721	0.2606	0.3728	0.7100	No
**3xTg-AD 12m**	0.065044, 0.087678, 0.096180, 0.113207, 0.156653	0.065044	0.156653	0.2471	0.4743	0.7100	No
**3xTg-AD 16m**	0.050189, 0.061574, 0.080724, 0.138954, 0.139538	0.050189	0.139538	0.1274	0.0065	0.7100	No

Table S2. PECAM-1 individual values and Dixon Q audit

Group	Sorted individual values	Minimum	Maximum	Qmin	Qmax	Qcrit alpha 0.05	Flagged for sensitivity
**Non-Tg 3m**	0.109505, 0.115091, 0.146077, 0.164649, 0.280604	0.109505	0.280604	0.0326	0.6777	0.7100	No
**Non-Tg 6m**	0.088580, 0.111088, 0.119150, 0.143516, 0.257703	0.088580	0.257703	0.1331	0.6752	0.7100	No
**Non-Tg 12m**	0.013729, 0.083420, 0.086238, 0.139065, 0.171733	0.013729	0.171733	0.4411	0.2068	0.7100	No
**Non-Tg 16m**	0.049108, 0.065720, 0.143529, 0.177989, 0.198513	0.049108	0.198513	0.1112	0.1374	0.7100	No
**3xTg-AD 3m**	0.053360, 0.099939, 0.161097, 0.199436, 0.280807	0.053360	0.280807	0.2048	0.3578	0.7100	No
**3xTg-AD 6m**	0.073459, 0.097499, 0.129218, 0.192408, 0.216783	0.073459	0.216783	0.1677	0.1701	0.7100	No
**3xTg-AD 12m**	0.009674, 0.074523, 0.099138, 0.125608, 0.156573	0.009674	0.156573	0.4415	0.2108	0.7100	No
**3xTg-AD 16m**	0.014003, 0.117459, 0.154750, 0.162579, 0.236325	0.014003	0.236325	0.4653	0.3317	0.7100	No

Table S3. Claudin-1 individual values and Dixon Q audit

Group	Sorted individual values	Minimum	Maximum	Qmin	Qmax	Qcrit alpha 0.05	Flagged for sensitivity
**Non-Tg 3m**	0.02892152, 0.11930765, 0.33777990, 0.36878494, 0.40813330	0.02892152	0.40813330	0.2384	0.1038	0.7100	No
**Non-Tg 6m**	0.14582041, 0.31415507, 0.40616969, 0.42988620, 0.68025470	0.14582041	0.68025470	0.3150	0.4685	0.7100	No
**Non-Tg 12m**	0.10705830, 0.15045070, 0.23114074, 0.23642320, 0.43425415	0.10705830	0.43425415	0.1326	0.6046	0.7100	No
**Non-Tg 16m**	0.02051940, 0.05487490, 0.20303692, 0.27265099, 0.37772963	0.02051940	0.37772963	0.0962	0.2942	0.7100	No
**3xTg-AD 3m**	0.02441130, 0.04822440, 0.27485538, 0.45073079, 0.54705327	0.02441130	0.54705327	0.0456	0.1843	0.7100	No
**3xTg-AD 6m**	0.02587720, 0.18778290, 0.43794222, 0.47212123, 0.56979475	0.02587720	0.56979475	0.2977	0.1796	0.7100	No
**3xTg-AD 12m**	0.02409810, 0.20439190, 0.23673210, 0.26438159, 0.47270422	0.02409810	0.47270422	0.4019	0.4644	0.7100	No
**3xTg-AD 16m**	0.02335780, 0.02649274, 0.17452640, 0.21569883, 0.42540428	0.02335780	0.42540428	0.0078	0.5216	0.7100	No

Table S4. Claudin-3 individual values and Dixon Q audit

Group	Sorted individual values	Minimum	Maximum	Qmin	Qmax	Qcrit alpha 0.05	Flagged for sensitivity
**Non-Tg 3m**	0.16606464, 0.35660560, 0.41838550, 0.50823308, 0.51364798	0.16606464	0.51364798	0.5482	0.0156	0.7100	No
**Non-Tg 6m**	0.31125630, 0.33233705, 0.41923460, 0.42001362, 0.49088210	0.31125630	0.49088210	0.1174	0.3945	0.7100	No
**Non-Tg 12m**	0.19768500, 0.21369389, 0.25413380, 0.34232418, 0.40963186	0.19768500	0.40963186	0.0755	0.3176	0.7100	No
**Non-Tg 16m**	0.04168120, 0.08112890, 0.26341831, 0.31075160, 0.36445538	0.04168120	0.36445538	0.1222	0.1664	0.7100	No

**3xTg-AD 3m**	0.04755350, 0.14372400, 0.24445824, 0.27112578, 0.83532417	0.04755350	0.83532417	0.1221	0.7162	0.7100	Maximum: 0.83532417
**3xTg-AD 6m**	0.12577420, 0.16737544, 0.36985180, 0.36998650, 0.90281789	0.12577420	0.90281789	0.0535	0.6857	0.7100	No
**3xTg-AD 12m**	0.09173622, 0.10993710, 0.34757728, 0.59645840, 0.92263038	0.09173622	0.92263038	0.0219	0.3926	0.7100	No
**3xTg-AD 16m**	0.05919730, 0.11322110, 0.38696532, 0.48418880, 1.13920058	0.05919730	1.13920058	0.0500	0.6065	0.7100	No

Table S5. Claudin-5 individual values and Dixon Q audit

Group	Sorted individual values	Minimum	Maximum	Qmin	Qmax	Qcrit alpha 0.05	Flagged for sensitivity
**Non-Tg 3m**	0.2135015, 0.3203396, 0.3282354, 0.3730637, 0.5085667	0.213502	0.508567	0.3621	0.4592	0.7100	No
**Non-Tg 6m**	0.1575638, 0.2297945, 0.3842375, 0.5656451, 0.638126	0.157564	0.638126	0.1503	0.1508	0.7100	No
**Non-Tg 12m**	0.1499216, 0.2052221, 0.2248967, 0.3378311, 0.4710886	0.149922	0.471089	0.1722	0.4149	0.7100	No
**Non-Tg 16m**	0.1416994, 0.1478721, 0.1769252, 0.5473559, 0.6991534	0.141699	0.699153	0.0111	0.2723	0.7100	No
**3xTg-AD 3m**	0.1169854, 0.1769216, 0.29424, 0.3130316, 0.4204129	0.116985	0.420413	0.1975	0.3539	0.7100	No
**3xTg-AD 6m**	0.2699403, 0.3063465, 0.3495188, 0.4893621, 0.5290357	0.26994	0.529036	0.1405	0.1531	0.7100	No
**3xTg-AD 12m**	0.04313396, 0.05648108, 0.1752912, 0.1966939, 0.5883524	0.043134	0.588352	0.0245	0.7184	0.7100	Maximum: 0.5883524
**3xTg-AD 16m**	0.02043732, 0.09834812, 0.1244902, 0.1636582, 0.2120623	0.0204373	0.212062	0.4066	0.2526	0.7100	No

Table S6. Occludin individual values and Dixon Q audit

Group	Sorted individual values	Minimum	Maximum	Qmin	Qmax	Qcrit alpha 0.05	Flagged for sensitivity
**Non-Tg 3m**	0.1328403, 0.2026129, 0.2109178, 0.2486553, 0.2792612	0.13284	0.279261	0.4765	0.2090	0.7100	No
**Non-Tg 6m**	0.1224633, 0.2227245, 0.2598169, 0.2988505, 0.4098478	0.122463	0.409848	0.3489	0.3862	0.7100	No
**Non-Tg 12m**	0.03279003, 0.06180537, 0.127071, 0.2306563, 0.3293293	0.03279	0.329329	0.0978	0.3327	0.7100	No
**Non-Tg 16m**	0.006588479, 0.01194414, 0.2197876, 0.2493499, 0.3475397	0.00658848	0.34754	0.0157	0.2880	0.7100	No
**3xTg-AD 3m**	0.01215266, 0.02530789, 0.02696914, 0.04104201, 0.114107	0.0121527	0.114107	0.1290	0.7166	0.7100	Maximum: 0.114107
**3xTg-AD 6m**	0.009762484, 0.0203676, 0.03204169, 0.08104701, 0.2744546	0.00976248	0.274455	0.0401	0.7307	0.7100	Maximum: 0.2744546
**3xTg-AD 12m**	0.009573739, 0.01135672, 0.01781994, 0.2035829, 0.2872207	0.00957374	0.287221	0.0064	0.3012	0.7100	No
**3xTg-AD 16m**	0.008395376, 0.01049767, 0.01418252, 0.1753826, 0.2165182	0.00839538	0.216518	0.0101	0.1977	0.7100	No

Table S7. VE-cadherin approximately 120 kDa individual values and Dixon Q audit

Group	Sorted individual values	Minimum	Maximum	Qmin	Qmax	Qcrit alpha 0.05	Flagged for sensitivity
**Non-Tg 3m**	0.020631, 0.064530, 0.082564, 0.219966, 0.242779	0.020631	0.242779	0.1976	0.1027	0.7100	No
**Non-Tg 6m**	0.066321, 0.124772, 0.143931, 0.154039, 0.162672	0.066321	0.162672	0.6066	0.0896	0.7100	No
**Non-Tg 12m**	0.013865, 0.103962, 0.110538, 0.249333, 0.418131	0.013865	0.418131	0.2229	0.4175	0.7100	No
**Non-Tg 16m**	0.012956, 0.078433, 0.089842, 0.417127, 0.460779	0.012956	0.460779	0.1462	0.0975	0.7100	No
**3xTg-AD 3m**	0.024010, 0.109680, 0.121975, 0.136292, 0.264390	0.024010	0.264390	0.3564	0.5329	0.7100	No
**3xTg-AD 6m**	0.041141, 0.101050, 0.177636, 0.187653, 0.257088	0.041141	0.257088	0.2774	0.3215	0.7100	No
**3xTg-AD 12m**	0.008464, 0.116592, 0.222428, 0.225005, 0.376477	0.008464	0.376477	0.2938	0.4116	0.7100	No
**3xTg-AD 16m**	0.165442, 0.183698, 0.193036, 0.203312, 0.246736	0.165442	0.246736	0.2246	0.5342	0.7100	No

Table S8. VE-cadherin approximately 90 kDa individual values and Dixon Q audit

Group	Sorted individual values	Minimum	Maximum	Qmin	Qmax	Qcrit alpha 0.05	Flagged for sensitivity
**Non-Tg 3m**	0.12433381, 0.21329336, 0.21874992, 0.27061206, 0.39018350	0.12433381	0.39018350	0.3346	0.4498	0.7100	No
**Non-Tg 6m**	0.11234781, 0.17128577, 0.21959964, 0.23691483, 0.41313588	0.11234781	0.41313588	0.1959	0.5859	0.7100	No
**Non-Tg 12m**	0.08551753, 0.12219024, 0.21855634, 0.37745251, 0.48592441	0.08551753	0.48592441	0.0916	0.2709	0.7100	No
**Non-Tg 16m**	0.05055067, 0.10589171, 0.25221543, 0.42826911, 0.49135342	0.05055067	0.49135342	0.1255	0.1431	0.7100	No

**3xTg-AD 3m**	0.18417111, 0.19161597, 0.23188650, 0.24898239, 0.55426847	0.18417111	0.55426847	0.0201	0.8249	0.7100	Maximum: 0.55426847
**3xTg-AD 6m**	0.20402396, 0.20771891, 0.25303881, 0.33864336, 0.42753140	0.20402396	0.42753140	0.0165	0.3977	0.7100	No
**3xTg-AD 12m**	0.19370807, 0.21078146, 0.25480069, 0.34520470, 0.46690589	0.19370807	0.46690589	0.0625	0.4455	0.7100	No
**3xTg-AD 16m**	0.26225681, 0.26940539, 0.33566477, 0.36463451, 0.48324256	0.26225681	0.48324256	0.0323	0.5367	0.7100	No

Table S9. pSRC/SRC individual values and Dixon Q audit

Group	Sorted individual values	Minimum	Maximum	Qmin	Qmax	Qcrit alpha 0.05	Flagged for sensitivity
**Non-Tg 3m**	0.435494750, 0.630479759, 0.630782685, 0.778625034, 3.080086292	0.435495	3.080086292	0.0737	0.8703	0.7100	Maximum: 3.080086292
**3xTg-AD 3m**	0.178528987, 0.629052546, 0.630801884, 0.630813973, 0.630880984	0.178529	0.630881	0.9960	0.0001	0.7100	Minimum: 0.178528987

Table S10. MMP-9-immunoreactive approximately 65 kDa band individual values and Dixon Q audit

Group	Sorted individual values	Minimum	Maximum	Qmin	Qmax	Qcrit alpha 0.05	Flagged for sensitivity
**Non-Tg 3m**	0.109299351, 0.285630810, 0.294126090, 0.323180640, 0.354545250	0.109299	0.354545	0.7190	0.1279	0.7100	Minimum: 0.109299351
**3xTg-AD 3m**	0.253328090, 0.312691610, 0.321030310, 0.339616030, 0.348738910	0.253328	0.348739	0.6222	0.0956	0.7100	No

Table S11. Observations flagged by Dixon Q (two-sided alpha = 0.05; Qcrit = 0.710) for sensitivity analyses

Outcome	Group	Tail	Exact value	Q calculated	Q critical	Use
**Claudin-3**	3xTg-AD 3m	Maximum	0.83532417	0.7162	0.710	Sensitivity only
**Claudin-5**	3xTg-AD 12m	Maximum	0.5883524	0.7184	0.710	Sensitivity only
**Occludin**	3xTg-AD 3m	Maximum	0.114107	0.7166	0.710	Sensitivity only
**Occludin**	3xTg-AD 6m	Maximum	0.2744546	0.7307	0.710	Sensitivity only
**VE-cadherin ~90 kDa**	3xTg-AD 3m	Maximum	0.55426847	0.8249	0.710	Sensitivity only
**pSRC/SRC**	Non-Tg 3m	Maximum	3.08008629	0.8703	0.710	Sensitivity only

**pSRC/SRC**	3xTg-AD 3m	Minimum	0.178528987	0.9960	0.710	Sensitivity only
**MMP-9-immunoreactive ~65 kDa band**	Non-Tg 3m	Minimum	0.109299351	0.7190	0.710	Sensitivity only

Table S12. Complete-data two-way ANOVA for the eight protein outcomes

Outcome	Scale	Effect	df1	df2	F	Exact p	Partial η²
**PDGFR-beta**	raw	Genotype	1	32	3.460	0.072098	0.098
**PDGFR-beta**	raw	Age	3	32	0.155	0.925811	0.014
**PDGFR-beta**	raw	Genotype x age	3	32	0.612	0.612434	0.054
**PECAM-1**	raw	Genotype	1	32	0.001	0.981307	0.000
**PECAM-1**	raw	Age	3	32	1.562	0.217776	0.128
**PECAM-1**	raw	Genotype x age	3	32	0.027	0.993927	0.003
**Claudin-1**	raw	Genotype	1	32	0.037	0.848341	0.001
**Claudin-1**	raw	Age	3	32	1.881	0.152603	0.150
**Claudin-1**	raw	Genotype x age	3	32	0.082	0.969472	0.008
**Claudin-3**	log10	Genotype	1	32	0.051	0.822433	0.002
**Claudin-3**	log10	Age	3	32	0.648	0.590222	0.057
**Claudin-3**	log10	Genotype x age	3	32	0.809	0.498111	0.071
**Claudin-5**	raw	Genotype	1	32	3.187	0.083706	0.091
**Claudin-5**	raw	Age	3	32	1.914	0.147082	0.152
**Claudin-5**	raw	Genotype x age	3	32	0.729	0.542147	0.064
**Occludin**	**raw**	**Genotype**	**1**	**32**	**12.446**	**0.001291**	**0.280**
**Occludin**	raw	Age	3	32	0.423	0.737625	0.038
**Occludin**	raw	Genotype x age	3	32	0.882	0.460732	0.076
**VE-cadherin 120 kDa**	raw	Genotype	1	32	0.027	0.869885	0.001
**VE-cadherin 120 kDa**	raw	Age	3	32	0.898	0.452912	0.078
**VE-cadherin 120 kDa**	raw	Genotype x age	3	32	0.039	0.989466	0.004
**VE-cadherin 90 kDa**	log10	Genotype	1	32	3.050	0.090337	0.087
**VE-cadherin 90 kDa**	log10	Age	3	32	0.027	0.993896	0.003
**VE-cadherin 90 kDa**	log10	Genotype x age	3	32	0.253	0.858741	0.023

Table S13. Benjamini-Hochberg FDR control across complete-data omnibus genotype effects

Outcome	Omnibus genotype p	BH q	Significant at q < 0.05
**PDGFR-beta**	0.072098	0.1807	No
**PECAM-1**	0.981307	0.9813	No
**Claudin-1**	0.848341	0.9813	No
**Claudin-3**	0.822433	0.981308	No
**Claudin-5**	0.083706	0.1807	No
**Occludin**	**0.001291**	**0.0103**	**Yes**
**VE-cadherin 120 kDa**	0.869885	0.9813	No
**VE-cadherin 90 kDa**	0.090337	0.1807	No

Table S14. Complete-data age-specific genotype contrasts with effect estimates

Outcome	Scale	Age	n Non-Tg/3xTg-AD	Mean difference Non-Tg minus 3xTg-AD (95% CI)	Hedges' g	Unadjusted p	Šídák p across 4 ages
**PDGFR-beta**	raw	3m	5/5	0.0603 (−0.0279, 0.1484)	0.878	0.1733	0.5329
**PDGFR-beta**	raw	6m	5/5	−0.0101 (−0.0983, 0.0780)	−0.157	0.8164	0.9989
**PDGFR-beta**	raw	12m	5/5	0.0597 (−0.0284, 0.1479)	0.628	0.1772	0.5416
**PDGFR-beta**	raw	16m	5/5	0.0511 (−0.0370, 0.1393)	0.722	0.2462	0.6771
**PECAM-1**	raw	3m	5/5	0.0043 (−0.0852, 0.0937)	0.048	0.9234	1.0000
**PECAM-1**	raw	6m	5/5	0.0021 (−0.0873, 0.0916)	0.030	0.9615	1.0000
**PECAM-1**	raw	12m	5/5	0.0057 (−0.0837, 0.0952)	0.089	0.8969	0.9999
**PECAM-1**	raw	16m	5/5	−0.0101 (−0.0995, 0.0794)	−0.122	0.8204	0.9990
**Claudin-1**	raw	3m	5/5	−0.0165 (−0.2497, 0.2168)	−0.073	0.8865	0.9998
**Claudin-1**	raw	6m	5/5	0.0566 (−0.1767, 0.2898)	0.243	0.6248	0.9802
**Claudin-1**	raw	12m	5/5	−0.0086 (−0.2419, 0.2247)	−0.054	0.9406	1.000
**Claudin-1**	raw	16m	5/5	0.0127 (−0.2206, 0.2459)	0.073	0.9126	0.999
**Claudin-3**	log10	3m	5/5	0.2466 (−0.2123, 0.7055)	0.635	0.2819	0.7341
**Claudin-3**	log10	6m	5/5	0.1073 (−0.3516, 0.5661)	0.396	0.6372	0.9827
**Claudin-3**	log10	12m	5/5	−0.0215 (−0.4804, 0.4374)	−0.059	0.9244	1.0000
**Claudin-3**	log10	16m	5/5	−0.2303 (−0.6892, 0.2285)	−0.446	0.3142	0.7789
**Claudin-5**	raw	3m	5/5	0.0844 (-0.1297, 0.2985)	0.673	0.4278	0.8928
**Claudin-5**	raw	6m	5/5	0.0062 (-0.2079, 0.2203)	0.034	0.9531	1.0000
**Claudin-5**	raw	12m	5/5	0.0658 (-0.1483, 0.2799)	0.329	0.5357	0.9535

**Claudin-5**	raw	16m	5/5	0.2188 (0.0047, 0.4329)	1.028	0.0454	0.1697
**Occludin**	**raw**	**3m**	**5/5**	**0.1709 (0.0316, 0.3103)**	**3.188**	**0.0178**	**0.0692**
**Occludin**	**raw**	**6m**	**5/5**	**0.1792 (0.0399, 0.3185)**	**1.503**	**0.0133**	**0.0523**
**Occludin**	raw	12m	5/5	0.0504 (-0.0889, 0.1897)	0.359	0.4664	0.9189
**Occludin**	raw	16m	5/5	0.0820 (-0.0573, 0.2214)	0.573	0.2391	0.6648
**VE-cadherin 120 kDa**	raw	3m	5/5	−0.0052 (−0.1593, 0.1489)	−0.050	0.9459	1.0000
**VE-cadherin 120 kDa**	raw	6m	5/5	−0.0226 (−0.1767, 0.1315)	−0.314	0.7674	0.9971
**VE-cadherin 120 kDa**	raw	12m	5/5	−0.0106 (−0.1647, 0.1435)	−0.065	0.8892	0.9998
**VE-cadherin 120 kDa**	raw	16m	5/5	0.0134 (−0.1407, 0.1675)	0.081	0.8607	0.9996
**VE-cadherin 90 kDa**	log10	3m	5/5	−0.0531 (−0.3576, 0.2513)	−0.256	0.7245	0.9942
**VE-cadherin 90 kDa**	log10	6m	5/5	−0.1149 (−0.4193, 0.1895)	−0.588	0.4477	0.9070
**VE-cadherin 90 kDa**	log10	12m	5/5	−0.1205 (−0.4249, 0.1840)	−0.433	0.4261	0.8915
**VE-cadherin 90 kDa**	log10	16m	5/5	−0.2335 (−0.5380, 0.0709)	−0.688	0.1280	0.4219

Table S15. Panel-level sensitivity analysis after excluding Dixon-flagged observations

Outcome	Excluded observation(s)	Genotype effect (partial η^2)	Exact p	BH q	q < 0.05
**PDGFR-beta**	None	F(1,32)=3.460; partial η^2=0.098	0.072098	0.1923	No
**PECAM-1**	None	F(1,32)=0.001; partial η^2=0.000	0.981307	0.9813	No
**Claudin-1**	None	F(1,32)=0.037; partial η^2=0.001	0.848341	0.9813	No
**Claudin-3**	3xTg-AD 3m maximum=0.83532417	F(1,31)=0.335; partial η^2=0.011	0.566714	0.906743	No
**Claudin-5**	3xTg-AD 12m maximum=0.5883524	F(1,31)=5.857; partial η^2=0.159	0.021569	0.0863	No
**Occludin**	3xTg-AD 3m maximum=0.114107; 3xTg-AD 6m maximum=0.2744546	**F(1,30)=16.424; partial η^2=0.354**	**0.000330**	**0.0026**	**Yes**
**VE-cadherin 120 kDa**	None	F(1,32)=0.027; partial η^2=0.001	0.869885	0.9813	No
**VE-cadherin 90 kDa**	3xTg-AD 3m maximum=0.55426847	F(1,31)=2.194; partial η^2=0.066	0.148635	0.2973	No

For sensitivity analyses that became unbalanced after Dixon-flagged observations were excluded, ordinary two-way ANOVA used Type III sums of squares. Complete-data analyses remained primary.

Table S16. Junction proteins age-specific contrasts in Dixon-excluded sensitivity analyses

Outcome	Age	n Non-Tg/3xTg-AD	Mean difference (95% CI)	Hedges' g	Unadjusted p	Šídák p	Adjusted significant
**Claudin-3**	3m	5/4	0.3981 (−0.0682, 0.8644)	1.295	0.0915	0.3188	No
**Claudin-3**	6m	5/5	0.1073 (−0.3324, 0.5469)	0.396	0.6223	0.9796	No
**Claudin-3**	12m	5/5	−0.0215 (−0.4612, 0.4181)	−0.059	0.9211	1.0000	No
**Claudin-3**	16m	5/5	−0.2303 (−0.6700, 0.2093)	−0.446	0.2935	0.7509	No
**Claudin-5**	3m	5/5	0.0844 (-0.1103, 0.2792)	0.673	0.3834	0.8555	No
**Claudin-5**	6m	5/5	0.0062 (-0.1885, 0.2010)	0.034	0.9484	1.0000	No
**Claudin-5**	12m	5/4	0.1599 (-0.0467, 0.3665)	1.296	0.1246	0.4126	No

**Claudin-5**	16m	5/5	0.2188 (0.0241, 0.4136)	1.028	0.0289	0.1107	No
**Occludin**	**3m**	**5/4**	**0.1885 (0.0464, 0.3305)**	**3.949**	**0.0110**	**0.0434**	**Yes**
**Occludin**	**6m**	**5/4**	**0.2269 (0.0849, 0.3690)**	**2.456**	**0.0028**	**0.0110**	**Yes**
**Occludin**	12m	5/5	0.0504 (-0.0835, 0.1844)	0.359	0.4480	0.9072	No
**Occludin**	16m	5/5	0.0820 (-0.0519, 0.2160)	0.573	0.2206	0.6309	No
**VE-cadherin 90 kDa**	3m	5/4	0.0302 (−0.2853, 0.3456)	0.188	0.8467	0.9994	No
**VE-cadherin 90 kDa**	6m	5/5	−0.1149 (−0.4123, 0.1825)	-0.588	0.4368	0.8994	No
**VE-cadherin 90 kDa**	12m	5/5	−0.1205 (−0.4179, 0.1770)	-0.433	0.4150	0.8829	No
**VE-cadherin 90 kDa**	16m	5/5	−0.2335 (−0.5310, 0.0639)	-0.688	0.1195	0.3989	No

Table S17. Complete-data and Dixon-excluded Welch t tests for pSRC/SRC and the approximately 65 kDa MMP-9-immunoreactive band

**Outcome**	**Analysis**	**n Non-Tg/3xTg-AD**	**Mean difference (95% CI)**	**Welch statistic**	**p**	**Hedges' g**
**pSRC/SRC**	Complete dataset	5/5	0.5711 (-0.7930, 1.9351)	t(4.27)=1.134	0.3163	0.648
**pSRC/SRC**	Dixon-excluded sensitivity	4/4	-0.0115 (-0.2355, 0.2124)	t(3.00)=-0.164	0.8801	-0.101
**MMP-9-immunoreactive ~65 kDa band**	Complete dataset	5/5	-0.0417 (-0.1584, 0.0750)	t(5.19)=-0.909	0.4037	-0.519
**MMP-9-immunoreactive ~65 kDa band**	Dixon-excluded sensitivity	4/5	-0.0007 (-0.0549, 0.0535)	t(6.96)=-0.031	0.9761	-0.018
